# Mitochondrial Function in Human Neuroblastoma Cells Is Up-Regulated and Protected by NQO1, a Plasma Membrane Redox Enzyme

**DOI:** 10.1371/journal.pone.0069030

**Published:** 2013-07-11

**Authors:** Jiyeong Kim, Su-Kyung Kim, Hwa-Kyung Kim, Mark P. Mattson, Dong-Hoon Hyun

**Affiliations:** 1 Department of Life Science, College of Natural Sciences, Ewha Womans University, Seoul, South Korea; 2 Laboratory of Neurosciences, National Institute on Aging Intramural Research Program, National Institutes of Health, Baltimore, Maryland, United States of America; Ludwig Maximilians University Munich, United States of America

## Abstract

**Background:**

Recent findings suggest that NADH-dependent enzymes of the plasma membrane redox system (PMRS) play roles in the maintenance of cell bioenergetics and oxidative state. Neurons and tumor cells exhibit differential vulnerability to oxidative and metabolic stress, with important implications for the development of therapeutic interventions that promote either cell survival (neurons) or death (cancer cells).

**Methods and Findings:**

Here we used human neuroblastoma cells with low or high levels of the PMRS enzyme NADH-quinone oxidoreductase 1 (NQO1) to investigate how the PMRS modulates mitochondrial functions and cell survival. Cells with elevated NQO1 levels exhibited higher levels of oxygen consumption and ATP production, and lower production of reactive oxygen species. Cells overexpressing NQO1 were more resistant to being damaged by the mitochondrial toxins rotenone and antimycin A, and exhibited less oxidative/nitrative damage and less apoptotic cell death. Cells with basal levels of NQO1 resulted in increased oxidative damage to proteins and cellular vulnerability to mitochondrial toxins. Thus, mitochondrial functions are enhanced and oxidative stress is reduced as a result of elevated PMRS activity, enabling cells to maintain redox homeostasis under conditions of metabolic and energetic stress.

**Conclusion:**

These findings suggest that NQO1 is a potential target for the development of therapeutic agents for either preventing neuronal degeneration or promoting the death of neural tumor cells.

## Introduction

Mitochondria are a hub for cellular energy metabolism because they produce the majority of ATP required for cell survival and maintenance of cell physiology [1], [2]. However, during oxidative phosphorylation, mitochondria generate free radicals, which can cause oxidative damage and mitochondrial dysfunction. Alterations in mitochondrial function and energy metabolism are believed to contribute to aging and age-related diseases [3], [4]. Defective activities of mitochondrial complexes I, II, III and IV have been identified in several major neurodegenerative diseases and to a lesser extent during normal aging [5], [6], [7], [8], and may result in reductions of ATP levels and ATP-dependent biochemical processes [9]. In addition, neurons are very vulnerable to acute oxidative and metabolic stresses that may occur under conditions of ischemia or hypoglycemia [1], [10]. It is therefore important to understand mechanisms by which neurons can maintain mitochondrial function under stressful conditions.

In contrast to postmitotic neurons, tumor cells are relatively resistant to metabolic and oxidative stress, in part because their mitochondria-mediated programmed cell death pathways are often disabled [11], [12]. Cellular energy metabolism is also typically altered in cancer cells such that glycolysis is increased and oxidative phosphorylation reduced [12].

The PMRS (plasma membrane redox system) can regulate redox homeostasis by promoting maintenance of a relatively high NAD^+^/NADH ratio [13]. In response to oxidative stress, electrons are transferred across the plasma membrane, from internal reductants such as NAD(P)H to external oxidants [14], [15], [16]. Coenzyme Q (CoQ), a key electron shuttle in the plasma membrane, can be reduced either by NAD(P)H-quinone oxidoreductase 1 (NQO1) [17], [18], [19] or by cytochrome b5 reductase [20], [21]. NQO1 is of particular interest because its expression is induced by Nrf2, a transcription factor involved in adaptive cellular responses to oxidative and metabolic stress, and NQO1 can be translocated to the inner surface of the plasma membrane under stressful conditions [22].

Recently, it was shown that neurons can be protected from oxidative and metabolic stresses through the activation of detoxifying enzymes including NQO1 in response to the activation of Nrf2 [23], [24]. Other reports indicate that altered NQO1 expression is related to the pathogenesis of Alzheimer°s disease (AD) [25], [26], and suggest a potential neuroprotective role for NQO1 in diseases involving metabolic and oxidative stresses including AD [27]. NQO1 can protect cultured cells against toxic insults by regulating PMRS activity [28]. However, it is not known whether NQO1 can modulate mitochondrial function.

In this study, we used human neuroblastoma cells with low or high NQO1 levels and assessed several mitochondrial functions in the absence or presence of mitochondrial inhibitors. We found that elevated levels of NQO1 enhance mitochondrial activity without causing increased production of reactive oxygen species (ROS), and protect cells against mitochondrial toxins, suggesting that mitochondrial bioenergetics is improved by the PMRS enzyme NQO1.

## Materials and Methods

### Cell Culture and Transfection

SH-SY5Y human neuroblastoma cells were cultured in DMEM medium supplemented with 10% fetal bovine serum (Invitrogen, Carlsbad, CA, USA), 100 IU/ml penicillin (Invitrogen) and 100 µg/ml streptomycin (Invitrogen) in a humidified 5% CO_2_/95% air atmosphere. The cells were transfected with pBE8 vector containing the full-length NQO1 cDNA (a generous gift from Alan Sartorelli at the Yale University School of Medicine) as described previously [29]. The cells were selected using G-418 and their relative levels of NQO1 were established by immunoblot analysis [28].

### Cell Viability Assays

Cell viability was determined by evaluating mitochondrial activity using 3-(4,5-dimethyl-thiazol-2-yl)-2,5-diphenyltetrazolium bromide (MTT) (Sigma, St. Louis, MO, USA) or membrane integrity using trypan blue staining [30], [31]. When cells reached 80% confluence, they were exposed to normal culture medium containing 100 µM rotenone (Sigma) or 100 µM antimycin A (Sigma) for 1–3 days. For the trypan blue assay, cells were trypsinized, washed twice with PBS (Invitrogen), and trypan blue dye solution was added and the number of dye-excluding cells counted on a hemocytometer in cells from 3 separate cultures. For the MTT assay, cell suspensions with equal numbers of cells were transferred into a 96-well plate for 1 day. MTT solution (10 µL) was then added to each well. The absorbance was read at 540 nm following incubation of the mixture for 1 hr at 37°C.

### Isolation of Mitochondria

Mitochondrial fractions were isolated from the cells by centrifugation, and kept at 4°C, as described previously with minor modifications [32]. Briefly, cells were washed with ice-cold PBS and homogenized in 10 mM Tris buffer (pH 7.6) with a protease inhibitor cocktail (1.5 mM) (Sigma). The homogenates were centrifuged at 600 g for 10 min at 4°C and then the supernatants were centrifuged again at 14,000 g for 10 min at 4°C. The resulting pellets were carefully removed and resuspended in the assay buffer.

### Confirmation of the Purity of the Mitochondrial Fractions

Immunoblot assays using enzyme markers for the cytosol and mitochondria were performed to establish the relative purity of the isolated mitochondrial fractions using antibodies against peroxiredoxin II (cytosol marker, 1∶2,000, Ab Frontier, South Korea), peroxiredoxin III (mitochondrial marker, 1∶2,000, Ab Frontier), Complex I (1∶2,000, Invitrogen), Complex II (1∶2,000, Abcam, Cambridge, MA, USA), Complex III (1∶2,000, Abcam), Complex IV (1∶2,000, Abcam), and prohibitin (1∶4,000, Santa Cruz Biotechnology, Santa Cruz, CA, USA). Briefly, the isolated mitochondrial fractions were lysed in PBS (pH 7.4) containing 5 µg/ml aprotinin, 5 µg/ml leupeptin, 5 µg/mL pepstastin A, and 0.1% Triton X-100, and placed on ice for 5 min. The lysates were centrifuged at 12,000 g for 10 min, and the supernatants were transferred into new Eppendorf tubes. Protein levels were measured using the Bradford reagent [33], and 10 µg of protein was separated by SDS-PAGE, as described previously [34], [35]. The separated proteins were transferred electrophoretically to a nitrocellulose membrane (Whatman GmBH, Dassel, Germany), which was then incubated with the primary antibodies. Immune complexes were detected with horseradish peroxidase-conjugated secondary antibodies and enhanced chemiluminescence reagents (1∶5,000, Ab Frontier).

### ATP Production Rate

Mitochondria were isolated from the cells and the ATP production rate (APR) was determined as previously described, with minor modifications [36]. Isolated mitochondria (10 µg) were suspended in reaction buffer A (0.1% BSA, 150 mM KCl, 0.1 mM MgCl_2_, 25 mM Tris-HCl, 10 mM potassium phosphate, pH 7.4) containing 160 µM diadenosine pentaphosphate, 1 mM pyruvate, 100 µM ADP, and either 5 mM NADH or 5 mM glutamate/malate or 5 mM succinate. To determine APR, luciferase assays were performed with buffer B (500 mM Tris-acetate, pH 7.75) containing 20 µg/ml luciferase (Invitrogen) and 0.8 mM D-luciferin (Invitrogen). The light emission was monitored using a luminometer (20/20^n^, Turner Biosystems, Sunnyvale, CA) for 5 min at 10 sec intervals. A standard curve for luminescence was made by using solutions containing increasing concentrations of ATP.

### Oxygen Consumption Rate

Oxygen consumption rate of the isolated mitochondria was monitored using an Instech Two Channel Fiber Optic Oxygen Monitor System (Instech Laboratories, Inc., Plymouth Meeting, PA, USA), as described previously [35]. Cell chambers were maintained at 37°C in a circulating water bath. All measurements were assessed with Fiber Optic Oxygen Monitor Operating Software and Slope Calculator Software (Instech Laboratories). Respiration rates were determined in 20 µg of each mitochondrial fraction in the presence of 5 mM NADH, 5 mM glutamate/malate or 5 mM succinate, and results were calculated as nmol oxygen consumed/min/mg protein. Activated oxygen consumption rate was measured following addition of ADP. Resting respiration rate was determined as the oxygen consumption rate under conditions where no inhibitors or uncouplers were administered to themitochondria. Non-mitochondrial oxygen consumption was determined as the respiration rate following incubation of cells with 100 µM potassium cyanide (KCN) (Fluka BioChemika, Bruchs, Germany).

### Production of Reactive Oxygen Species in the Mitochondria

Mitochondrial fractions isolated freshly from isolated freshly were analyzed. Hydrogen peroxide (H_2_O_2_) released from the mitochondria was measured using the fluorescent dye Amplex Red (Molecular Probes, Eugene, OR, USA) which reacts with H_2_O_2_ with a 1∶1 stoichiometry in the presence of horseradish peroxidase, and thereby produces the highly fluorescent chemical resorufin [37], [38], [39], [40]. Briefly, a reaction buffer (50 mM Tris, pH 7.4) was supplemented with 5 µM Amplex Red, 0.5 U/ml HRP and 20 U/mL Cu,Zn-superoxide dismutase to prevent the auto-oxidation of Amplex Red and interference with quantitative assessment of low rates of H_2_O_2_ production [41]. The supplemented buffers were preincubated at 37°C and mitochondrial fractions and electron donors (5 mM succinate or 5 mM glycerol 3-phosphate) were added to the reaction mixture. The detection of H_2_O_2_ in mitochondrial suspensions (0.1 mg protein/mL) was recorded as an increase in Amplex Red fluorescence using excitation and emission wavelengths of 560 and 590 nm, respectively. The response of Amplex Red to H_2_O_2_ was calibrated by incubation with known amounts of H_2_O_2_ solution from 100 to 1000 pmol. The concentration of commercial 30% H_2_O_2_ solution was calculated from light absorbance at 240 nm using E^240^ mM = 43.6 cm^–1^ and the stock solution was diluted to 100 µM with water and used for calibration immediately.

### Activities of Mitochondrial Complexes I and II

Mitochondrial complex I and II activities were assessed using decylubiquinone and dichloroindolphenol (DCIP), as described earlier [42]. Briefly, 10 µg of the isolated mitochondrial fractions were preincubated in the reaction buffer for complex I (70 µM decylubiquinone, 60 µM DCIP, 1 µM antimycin A, 0.35% BSA, 25 mM potassium phosphate, pH 7.4) or complex II (70 µM decylubiquinone, 60 µM DCIP, 1 µM antimycin A, 50 µM rotenone, 500 µM EDTA, 200 µM ATP, 0.1% BSA, 80 mM potassium phosphate, pH 7.4) at 37°C. The reaction was initiated by the addition of substrates (complex I: 5 mM NADH or 5 mM glutamate/malate and complex II: 5 mM succinate and 300 µM potassium cyanide) to the mixture and absorbance was read at 600 nm for 5 min with 20 sec intervals.

In the case of the inhibition study, cells were cultured in the presence of 50 and 100 µM rotenone for 6 hr and the mitochondrial fractions were isolated and used for the assays. Experiments using the mitochondrial fractions isolated from the cells cultured under normal conditions were also performed for the assays and then rotenone was added to the reaction mixture at lower concentrations (10 and 100 nM rotenone).

### NAD(P)^+^/NAD(P)H Ratio

NAD^+^ and NADH levels were determined separately using a NAD/NADH Quantitation Kit (BioVision, Mountain View, CA). Briefly, in order to measure total levels of NAD^+^ and NADH, lysates were transferred into a 96-well plate, and 100 µl NAD^+^ cycling buffer and 2 µl NAD^+^cycling enzyme mix were added. The mixtures were incubated at room temperature for 5 min to convert NAD^+^ to NADH, and NADH developer was then added to each well. The plate was incubated for 2 h, and absorbance was read at 450 nm. To measure NADH, NAD^+^ was decomposed by heating the lysates at 60°C for 30 min, followed by the same procedures described above. A standard curve was generated by serial dilution of standard NADH solution and the NAD^+^/NADH ratio was calculated as [(NADtotal–NADH)/NADH].

### Turnover Rate of p53

Cells were cultured in the presence of 0.5 µM cycloheximide for an appropriate time to prevent further protein synthesis. Then cells were lysed for immunoblot assays using an antibody against the tumor suppressor protein p53.

### Determination of Levels of Oxidative Stress Markers

Lipid peroxidation levels were assessed using the 8-Isoprostane Assay Kit (OxisResearch, Portland, OR, USA). Briefly, following exposure to each of the mitochondrial toxins, cells were lysed and cell extracts (100 µL) were added to a 96-well plate and incubated with 100 µL horseradish peroxidase-conjugated antibody at room temperature for 1 hr; 200 µL substrate was added to the plate and it was incubated for 30 min. Absorbance was read at 450 nm after stopping the reaction by adding 50 µL 3 M sulfuric acid. Protein carbonyl content was determined as described previously [43], except the final cell extracts were dissolved in 1 ml of 6 M guanidinium hydrochloride. Carbonyl content was calculated as nmol/mg protein [44]. Measurement of protein-bound nitrotyrosine content of isolated plasma membranes was performed using the Nitrotyrosine Assay Kit (OxisResearch).

### Evaluation of Apoptosis

Following exposure to rotenone or antimycin A for 6, 12, 18 and 24 hr, cells were lysed and a cleaved form of PARP was identified by immunoblot analysis using a PARP antibody (1∶2,000 dilution, Cell Signaling, Danvers, MA). Cell membrane damage (propidium iodide-positive cells) and chromatin condensation were also assessed using propidium iodide and Hoechst 33258, as described previously [30], [31].

### Statistical Analysis

Statistical differences were determined by one-way ANOVA, and multiple comparisons were performed with a post-hoc Bonferroni t-test.

## Results

Previously, we demonstrated that cells overexpressing NQO1 exhibited relative resistance to being damaged by KCN [28]. Here we found that MTT-reduction activity in NQO1 transfectants was higher than that in control cells under normal culture conditions (Fig. S1). These findings suggested that mitochondrial activity is influenced by the PMRS enzyme, NQO1.

### Mitochondria from Cells with Elevated NQO1 Levels Exhibit Increased ATP Production and Oxygen Consumption

**Figure 1:**
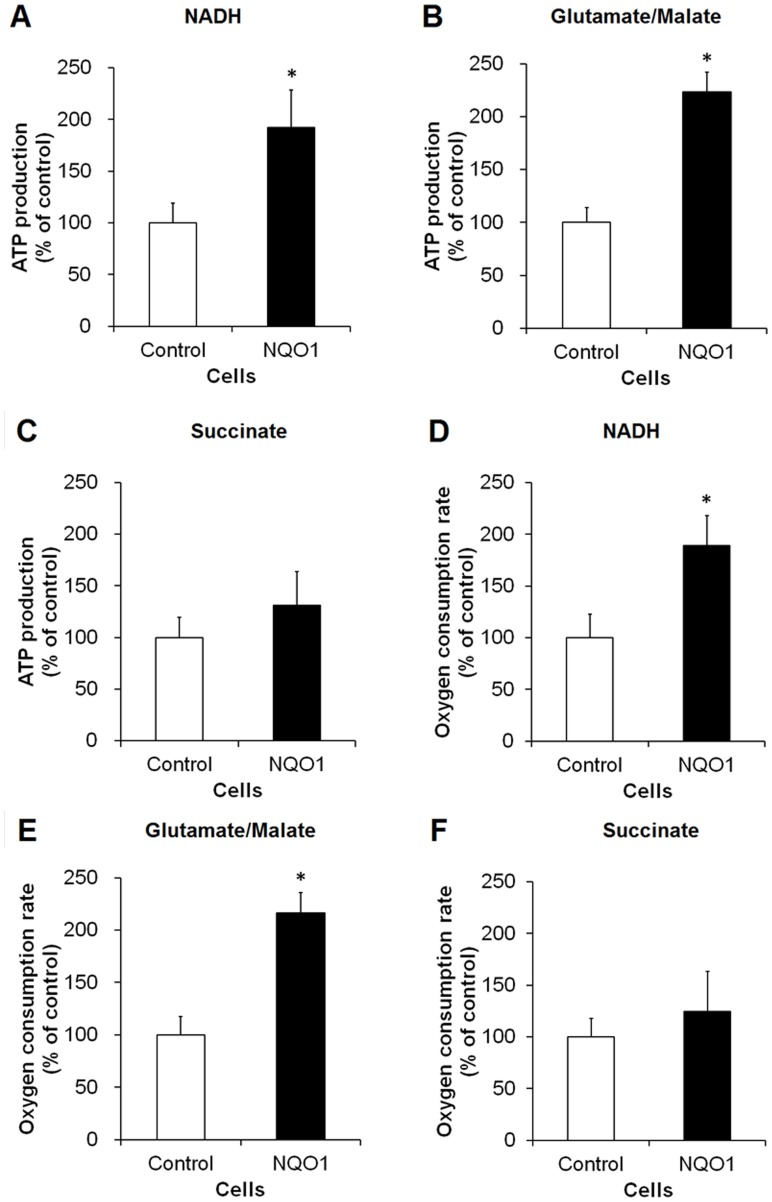
ATP production and oxygen consumption are increased by overexpression of NQO1. Intact mitochondria were prepared and ATP production and oxygen consumption were assessed in the presence of electron donors. (A–C) ATP production rates in the presence of NADH (A), glutamate/malate (B) and succinate (C). (D–F) Oxygen consumption rates in the presence of NADH (D), glutamate/malate (E) and succinate (F). Values are the mean ± SEM (n = 6). *p*<*0.01 compared with the value for untransfected control cells.

In order to determine whether ATP synthesis in the mitochondria was affected by NQO1 levels, mitochondrial ATP production rate (APR) was measured by a luciferase assay. Mitochondrial APR was significantly enhanced by overexpression of NQO1 in the presence of NADH or glutamate/malate (p<0.01) (Fig. 1A and B). Oxygen consumption rate was also elevated in NQO1 transfectants compared with control cells in the presence of NADH or glutamate/malate (p<0.01) (Fig. 1D and E). Activated oxygen consumption rate (state 3 respiration) was also significantly increased when the electron donors were added (p<0.01) (Fig. S2C and D). APR and oxygen consumption rate were slightly higher in NQO1 transfectants treated with succinate (Fig. 1C and F); however, they were not significant.

### Cells Overexpressing NQO1 Exhibit Enhanced Activities of Mitochondrial Electron Transport Chain Complexes and Reduced ROS Production

**Figure 2:**
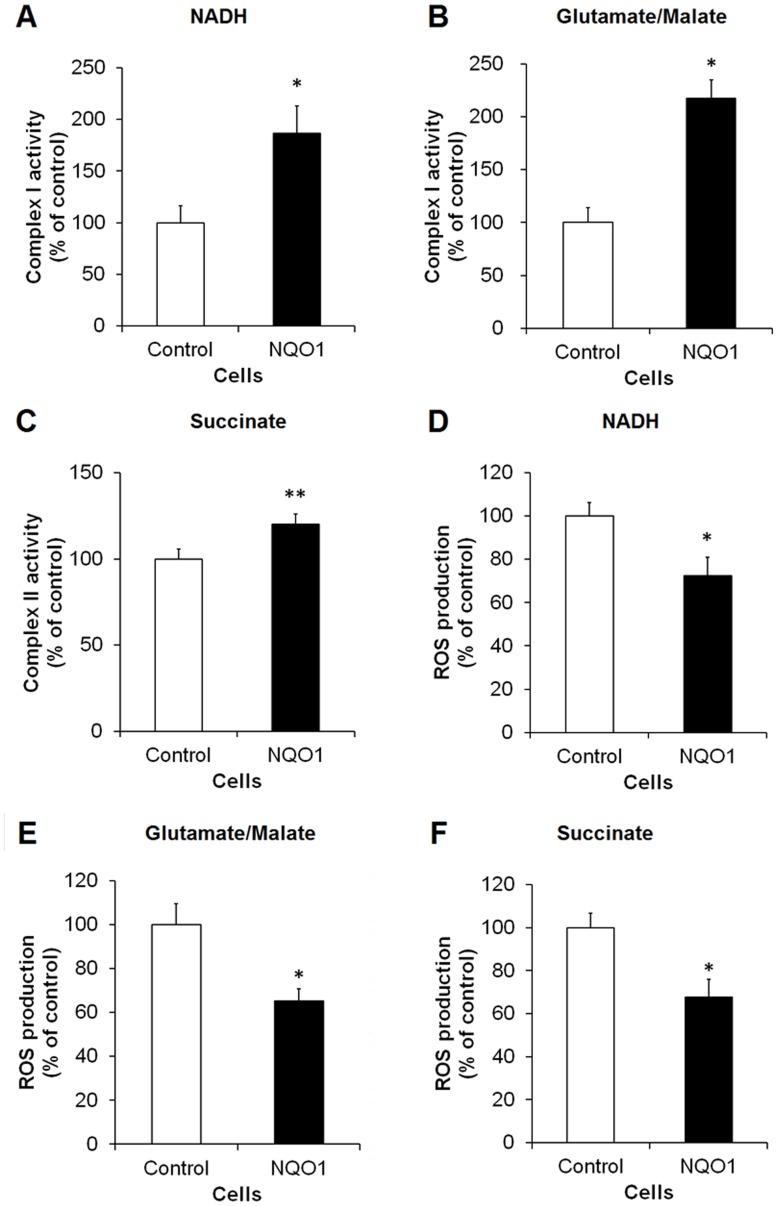
Activities of mitochondrial complexes I and II are elevated and production of ROS are reduced in human neuroblastoma cells overexpressing NQO1. Cells were cultured and then mitochondrial fractions were isolated by centrifugal fractionation. Mitochondrial complex activities were measured using appropriate electron donors. Complex I activity in the presence of NADH (A) and glutamate/malate (B). Complex II activity in the presence of succinate (C). ROS production in the presence of NADH (D), glutamate/malate (E) and succinate (F). Values are the mean ± SEM (n = 6). *p*<*0.01, **p<0.05 compared with the value for untransfected control cells.

To further elucidate the mechanism by which NQO1 influences mitochondrial function in human neural tumor cells, we measured activities of mitochondrial complexes I and II in cells expressing high or lower levels of NQO1. Mitochondrial complex I activity was significantly elevated in NQO1 transfectants compared to control cells (p<0.01) (Fig. 2A and B). Complex II activity was also higher in cells overexpressing NQO1 compared to control cells under normal culture conditions (p<0.05) (Fig. 2C). Interestingly, however, the production of ROS (in the presence of either NADH or glutamate/malate or succinate) was significantly lower in cells with elevated NQO1 levels compared to cells with a lower NQO1 level (p<0.01) (Fig. 2D, E and F).

### NQO1 Enhances Cellular Energetics and Stabilizes p53

**Figure 3:**
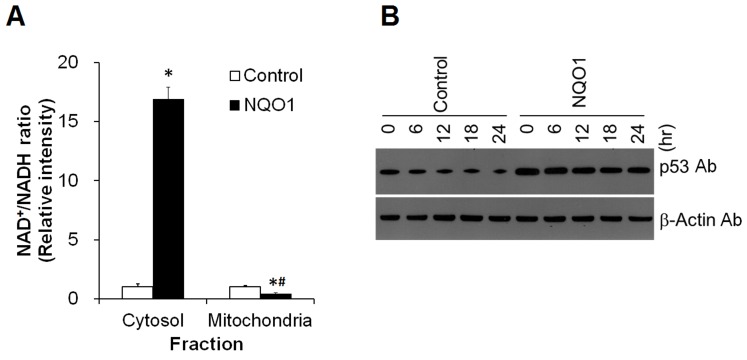
The cytoplasmic NAD^+^/NADH ratio and levels of p53 are elevated in cells overexpressing NQO1. (A) The NAD^+^/NADH ratio was measured using a NAD/NADH Quantitation Kit. Values are the mean ± SEM (n = 6). *p*<*0.01 compared with the value for untransfected control cells under normal culture conditions. ^#^ p<0.01 compared with the values in the mitochondrial fractions between untransfected control and NQO1-transfected cells. (B) p53 levels were assessed following treatment with cycloheximide for 6, 12, 18 and 24 hr.

The increased ATP production and oxygen consumption of cells overexpressing NQO1 suggests that NQO1 in the plasma membrane enhances cellular bioenergetics. To further characterize the effects of NQO1 on bioenergetics we measured levels of NAD^+^ and NADH in neuroblastoma cells with low or elevated levels of NQO1. The NAD^+^/NADH ratio in the cytosol of cells overexpressing NQO1 was approximately 10-fold greater than the NAD^+^/NADH ratio of control cells with lower NQO1 levels (p<0.01) (Fig. 3A). However, the NAD^+^/NADH ratio in the mitochondria of NQO1 transfectants was significantly decreased (p<0.01), by approximately 50% compared to control cells. Levels of p53 were highly expressed in cells overexpressing NQO1 (Fig. 3B). Following treatment with cycloheximide, an inhibitor of protein synthesis, turnover rate of p53 was reduced in cells with elevated NQO1 levels.

### NQO1 Protects Neuroblastoma Cells from Mitochondrial Toxins

**Figure 4:**
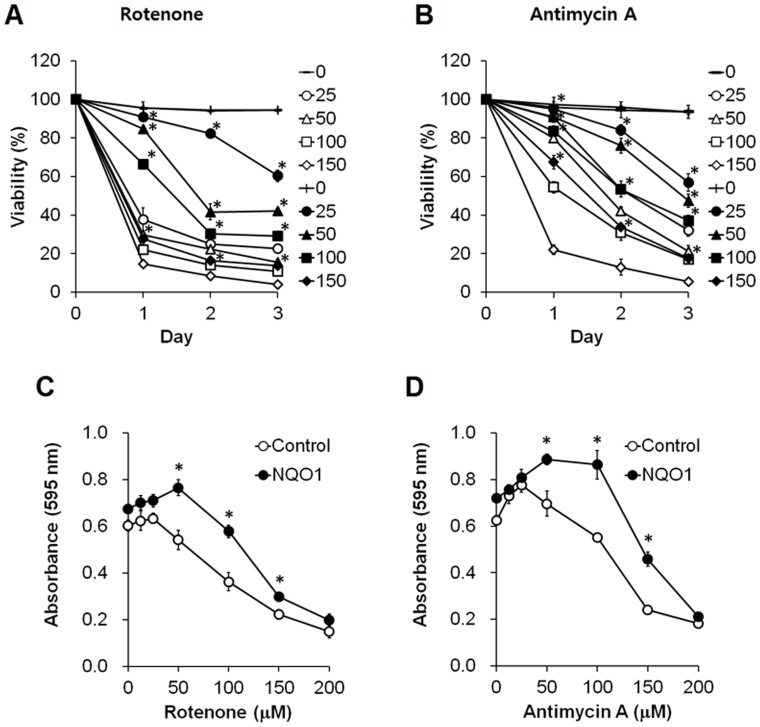
Neuroblastoma cells overexpressing NQO1 exhibit reduced vulnerability to mitochondrial toxins. Cells were exposed to the indicated concentrations (micromolar) of rotenone (A and C) or antimycin A (B and D) for the indicated time periods (A and B) or for 24 hr (C and D) and cell viability was measured by trypan blue exclusion (A and B, ○: control, •: NQO1) and MTT-reduction assay (C and D). Values are the mean ± SEM (n = 6). *p*<*0.01 compared to untransfected cells exposed to the same concentration of the toxin.

We performed cell viability experiments with both control and NQO1-overexpressing cell lines following exposure to mitochondrial toxins (the complex I inhibitor rotenone, and the complex III inhibitor antimycin A), which are known to induce ROS production. Following treatment with rotenone, cell viability was significantly decreased in the control cells with basal levels of NQO1 in a concentration-dependent manner (Fig. 4A and C). In contrast, cells overexpressing NQO1 were significantly more resistant to rotenone across the range of concentrations tested (p<0.01). Antimycin A reduced the viability of the cells with basal levels of NQO1 in a dose-dependent manner, and cells with elevated levels of NQO1 were significantly less vulnerable to the complex III inhibitor compared with the control cells (p<0.01) (Fig. 4B and D).

**Figure 5:**
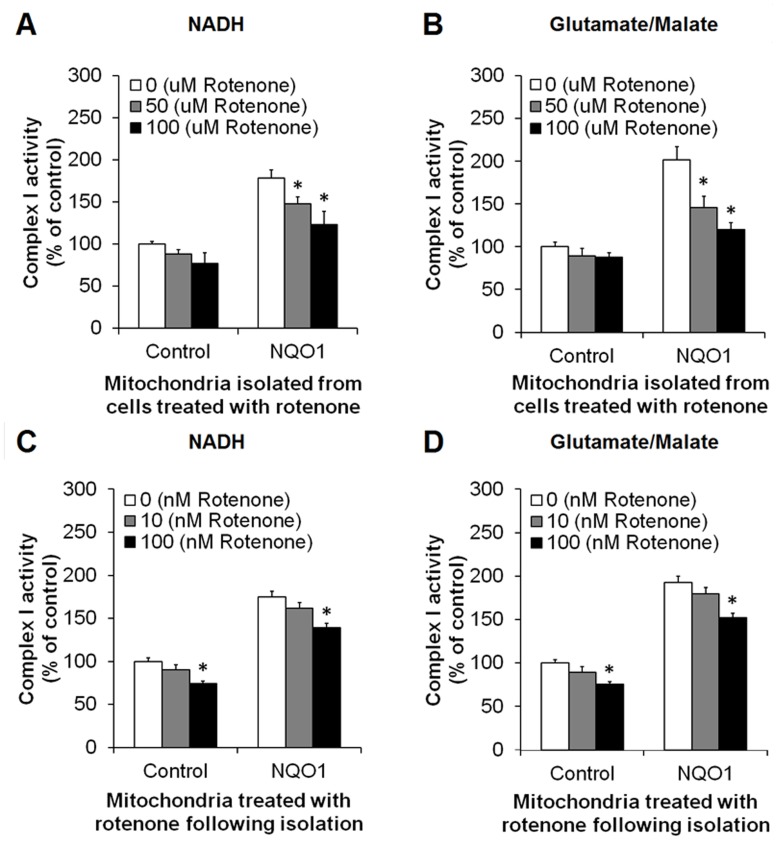
Complex I activity is increased in cells with elevated NQO1 levels and is decreased by rotenone. Cells were cultured in the presence of 50 and 100 µM rotenone for 6 hr and the mitochondrial fractions were isolated and used for the complex I assay using 5 mM NADH (A) or 5 mM glutamate/malate (B). The mitochondrial fractions were separated from cells cultured under normal culture conditions and the complex I assay was performed in the presence of 10 and 100 nM rotenone using 5 mM NADH (C) or 5 mM glutamate/malate (D). Values are the mean ± SEM (n = 6). *p*<*0.01 compared with the value for untransfected cells and to NQO1-overexpressing cells not exposed to rotentone.

The complex I activity in cells overexpressing NQO1 was significantly higher when the mitochondria were incubated in the presence of either NADH (Fig. 5A) or glutamate/malate (Fig. 5B). As expected, complex I activity in cells overexpressing NQO1 and treated with either NADH or glutamate/malate was significantly reduced when cells were exposed to rotenone (p<0.01) (Fig. 5A and B). Complex I activity in control cells treated with rotenone was also decreased following exposure to rotenone. However, it was less affected by the inhibitor than NQO1 transfectants (Fig. 5A and B). Similar patterns were also shown when the mitochondria were exposed to rotenone following isolation (Fig. 5C and D).

### NQO1 Attenuates Levels of Oxidative Protein Damage

**Figure 6:**
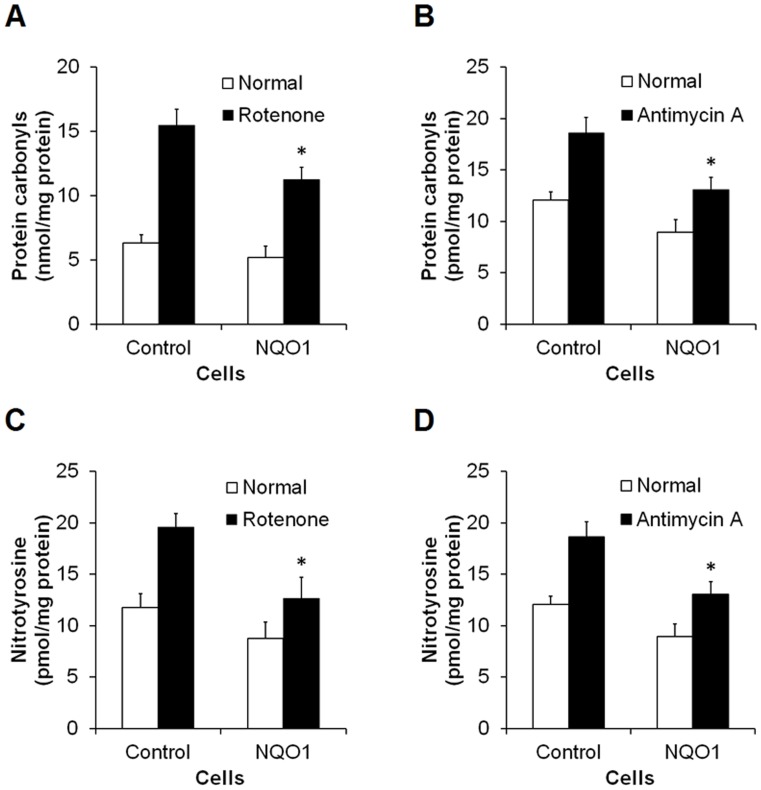
Levels of oxidative damage to lipids and proteins are attenuated in cells with elevated NQO1 levels compared with control cells. Cell extracts were used to measure levels of protein carbonyls (A and B) and nitrotyrosine (C and D) following exposure to 50 µM rotenone (A and C) or 100 µM antimycin A (B and D) for 24 hr. Values are the mean ± SEM (n = 6). *p*<*0.01 compared with the value for untransfected cells exposed to rotenone or antimycin A.

Levels of protein carbonyls, a biomarker for protein oxidation, were slightly lower in NQO1 transfectants compared with control cells under normal culture conditions. Treatment with rotenone dramatically increased levels of protein carbonyls in control cells (p<0.01) (Fig. 6A), and the rotenone-induced generation of protein carbonyls was significantly attenuated in cells with elevated NQO1 levels (p<0.01). Similarly, levels of nitrotyrosine, a marker of protein nitration, were significantly increased following the addition of rotenone (p<0.01), and this effect of rotenone was significantly attenuated in cells with high levels of NQO1 (p<0.01) (Fig. 6C). Antimycin A also induced increased levels of protein carbonyls (Fig. 6B) and 3-nitrotyrosine (Fig. 6D). Levels of both types of oxidative protein damage induced by antimycin were significantly lower in cells overexpressing NQO1 compared with control cells (p<0.01).

### Apoptosis is Reduced in Neuroblastoma Cells with Elevated Levels of NQO1

**Figure 7:**
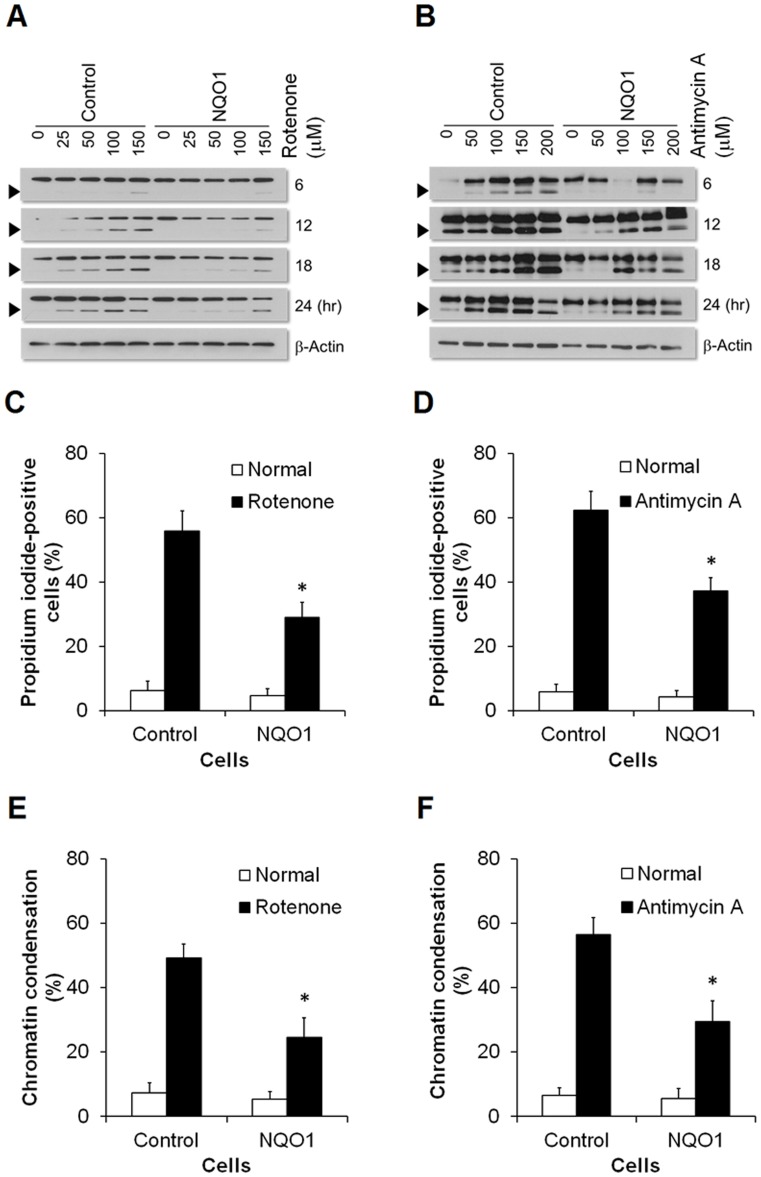
Neuroblastoma cells with elevated NQO1 levels exhibit resistance to apoptisis induced by mitochondrial toxins. Following exposure to rotenone (A) or antimycin A (B), cells were lysed and the levels of the cleaved form of PARP were determined by immunoblot analysis. Three independent experiments were performed and representative blots are shown. Levels of cell membrane permeabilization (propidium iodide-positive cells) (C and D) and chromatin condensation (E and F) were also measured after the addition of 50 µM rotenone (C and E) or 100 µM antimycin A (D and F) for 24 hr. Values are the mean ± SEM (n = 6). *p*<*0.01 compared with the value for untransfected control cells exposed to rotenone or antimycin A.

Cleavage of poly ADP ribose polymerase (PARP) was monitored as a cell death marker in in control and NQO1 overexpressing cells exposed to mitochondrial toxins. Addition of rotenone induced a cleaved form of PARP in a time-dependent and dose-dependent manner (Fig. 7A). Accumulation of the cleaved form of PARP occurred faster in control cells than in cells overexpressing NQO1. At the same concentration of rotenone, the appearance of propidium iodide-positive cells (Fig. 7C) and chromatin condensation (Fig. 7E) were delayed in cells overexpressing NQO1 compared with control cells. A similar resistance to apoptosis was evident in cells with elevated levels of NQO1 when antimycin A was used to trigger apoptosis (Fig. 7B, D and F).

## Discussion

NQO1 expression is known to be increased in response to energetic and oxidative stress. Recently, it has shown that the NQO1 substrates CoQ_1_ and idebenone could restore cellular ATP levels when mitochondrial complex I is inhibited [45]. We found that NQO1 influences several aspects of mitochondrial function including: elevation of mitochondrial complex I activity; increased ATP production; maintenance of an elevated NAD^+^/NADH ratio; and decreased ROS production. These effects of NQO1 on mitochondrial function would be expected to promote maintenance of cellular energy levels and reduce oxidative damage in cells under conditions of metabolic and oxidative stress. Indeed, we found that neuroblastoma cells overexpressing NQO1 were more resistant to being killed by the mitochondrial toxins rotenone and antimycin A.

Hydrocytyrosol, an inducer of Nrf2, can stimulate the expression of detoxifying enzymes including NQO1 and enhances mitochondrial biogenesis [46]. Other inducers of NQO1 expression, such as 3H-1,2-dithiole-3-thione and sulforaphane can protect cells against oxidative stress [47], [48], [49]. By stimulating the Nrf2 pathway, sulforaphane induces NQO1 expression and may thereby preserve mitochondrial function and protect mitochondria from toxic insults in some cells. Consistent with a role for NQO1 in cell survival, dicoumarol, a competitive inhibitor of NQO1, inhibits mitochondrial electron transport and increases the production of O_2_^−^, resulting in decreased cell proliferation [50], [51]. In some cells, on the other hand, NQO1 promotes cell death. For example, NQO1 causes apoptosis of non-small-cell lung cancer cells [52], suggesting unconventional roles for NQO1 in some types of cancer cells. The biological actions of NQO1 may therefore depend on the cell type and the metabolic/redox states of the cells.

As a result of the activity of the electron transport chain, the more ATP mitochondria produce, the more ROS they generate [2]. However, we found that overexpression of NQO1 results in decreased mitochondrial ROS levels in human neuroblastoma cells, even though they produce more ATP via increased activity of mitochondrial electron transport chain complexes compared to control cells under normal culture conditions. Interestingly, we found that a greater portion of complex I activity in NQO1 transfectants was reduced by addition of the complex I inhibitor, rotenone, compared with the control cells exposed to rotenone. There are at least four possibilities to explain these results. First, this might be causally linked to increased QH2/Q- and NAD/NADH-levels. Second, ROS generated during oxidative phosphorylation can be scavenged by NQO1 directly as NQO1 can reduce the oxidized form of CoQ. An alternative explanation is that, the effects are driven by uncoupling, caused by the generation of a cytosolic-mitochondrial electron shuttle [45], with an apparent higher complex I activity resulting, in part, from the low specificity of this assay. However, it is very likely that overexpressed NQO1 is not involved in reduction of CoQ by the electron shuttling because no NQO1 protein was detected in the isolated mitochondrial fractions (Fig. S2A). Third, ROS may also be neutralized by other mitochondrial antioxidant enzymes, some of which can be induced by the Nrf2 pathway [47], [48], [49]. Fourth, the mitochondria in cells with higher levels of NQO1 may generate ATP more efficiently without causing further leakage of electrons during the transfer of electrons from complex I or II to complex III.

ROS can be generated spontaneously in mitochondrial complexes involved in redox reactions, especially one-electron transfer via transition metals and semi-ubiquinone [53], [54]. Considerable evidence suggests that semiquinones linked to complexes I and III are responsible for the production of O_2_^−^ during oxidative phosphorylation [54], [55]. Many researchers measured ROS levels in the presence of specific inhibitors (e.g. rotenone, antimycin A) and in pathological conditions (e.g. hypoxia) [56], [57]. Previous studies showed that succinate and glutamate/malate are the best electron donors for mitochondria [58]. We found that cells with elevated NQO1 levels exhibited attenuated ROS production in mitochondria and an elevated NAD^+^/NADH ratio, suggesting that ROS can be neutralized by NQO1. Instead, we found that proteins and lipids in NQO1 transfectants were more resistant to oxidative/nitrative stress following treatment with either rotenone or antimycin A.

Human neuroblastoma cells including SH-SY5Y cell lines have been widely used as a complement to primary neuronal cultures because they have some common features of differentiated neurons [59]. For example, SH-SY5Y cells have been used as a study model of AD [60], [61], [62] and Parkinson’s disease (PD) [63], [64], [65]. We found that human neuroblastoma cells overexpressing NQO1 were relatively resistant to being killed by mitochondrial toxins as indicated by reduced PARP cleavage, cell shrinkage and chromatin condensation. These data are consistent with lower production of ROS, and lower levels of oxidative/nitrative damage in cells with elevated NQO1 levels. In fact, impaired energy metabolism and increased oxidative damage are known to contribute to degeneration of neurons in a variety of neurodegenerative diseases. Previously, we showed that normal aging is associated with reduced levels of NQO1 and other PMRS enzymes in brain cells [34]. Therefore, our findings suggest that NQO1 may play a key role in protecting neurons against oxidative stress and energy failure during aging and in neurodegenerative disorders, consistent with a previous result [66]. The present findings suggest that possibility that, by enhancing mitochondrial bioenergetics and reducing oxidative stress, up-regulation of NQO1 might protect neurons against pathogenic processes in neurodegenerative diseases including AD and PD.

Finally, our findings suggest that NQO1 is a target for the development of therapeutic interventions for neuroblastoma, and possibly other types of neural tumors. We found that overexpression of NQO1 prevents apoptosis of neuroblastoma cells which suggests that inhibition of NQO1 would enhance death of these cells. Consistent with the latter possibility, levels of NQO1 are elevated in glioblastoma cells and may protect them against apoptosis [67], and genetic deletion of NQO1 renders keratinocytes vulnerable to apoptosis [68]. On the other hand, activation of NQO1 can promote the death of some types of non-neural tumor cells. For example, it was reported that dicoumarol and other more specific inhibitors of NQO1 reduce the death of several types of cancer cells [69], and NQO1-deficient mice exhibit enhanced susceptibility to skin cancers [70]. Further work will be required to develop tumor type-specific NQO1-modifying agents for therapeutic intervention in cancers.

## Supporting Information

Figure S1 The same number of control and NQO1 transfected cells were plated onto 96-well plates. The next day, mitochondrial activity was assessed using a MTT-reduction assay. Values are the mean ± SEM (n = 6). *p<0.01 compared with the value for untransfected control cells under normal culture conditions.
(TIF)

Figure S2 Isolated mitochondrial fractions were assessed by immunoblot analysis using markers of the cytosol (peroxiredoxin II) or mitochondrial proteins (peroxiredoxin III, complexes I/II/III/IV and prohibitin). The fractions were also used to measure oxygen consumption rate (state3) in the presence of ADP.
(TIF)

## References

[1] Mattson MP, Gleichmann M, Cheng A (2008) Mitochondria in neuroplasticity and neurological disorders. Neuron 60: 748–766. 19081372 10.1016/j.neuron.2008.10.010 PMC2692277

[2] Murphy MP (2009) How mitochondria produce reactive oxygen species. Biochem J 417: 1–13. 19061483 10.1042/BJ20081386 PMC2605959

[3] Luft R, Landau BR (1995) Mitochondrial medicine. J Intern Med 238: 405–421. 7595180 10.1111/j.1365-2796.1995.tb01218.x

[4] Kim JA, Wei Y, Sowers JR (2008) Role of mitochondrial dysfunction in insulin resistance. Circ Res 102: 401–414. 18309108 10.1161/CIRCRESAHA.107.165472 PMC2963150

[5] Lesnefsky EJ, Slabe TJ, Stoll MS, Minkler PE, Hoppel CL (2001) Myocardial ischemia selectively depletes cardiolipin in rabbit heart subsarcolemmal mitochondria. Am J Physiol Heart Circ Physiol 280: H2770–2778. 11356635 10.1152/ajpheart.2001.280.6.H2770

[6] Menzies FM, Ince PG, Shaw PJ (2002) Mitochondrial involvement in amyotrophic lateral sclerosis. Neurochem Int 40: 543–551. 11850111 10.1016/s0197-0186(01)00125-5

[7] Schapira AH, Gu M, Taanman JW, Tabrizi SJ, Seaton T, et al (1998) Mitochondria in the etiology and pathogenesis of Parkinson’s disease. Ann Neurol 44: S89–98. 9749579 10.1002/ana.410440714

[8] Wang J, Xiong S, Xie C, Markesbery WR, Lovell MA (2005) Increased oxidative damage in nuclear and mitochondrial DNA in Alzheimer’s disease. J Neurochem 93: 953–962. 15857398 10.1111/j.1471-4159.2005.03053.x

[9] Schapira AH (1998) Mitochondrial dysfunction in neurodegenerative disorders. Biochim Biophys Acta 1366: 225–233. 9714816 10.1016/s0005-2728(98)00115-7

[10] Sims NR (1992) Energy metabolism and selective neuronal vulnerability following global cerebral ischemia. Neurochem Res 17: 923–931. 1407279 10.1007/BF00993269

[11] Griguer CE, Oliva CR (2011) Bioenergetics pathways and therapeutic resistance in gliomas: emerging role of mitochondria. Curr Pharm Des 17: 2421–2427. 21827418 10.2174/138161211797249251

[12] Kroemer G, Pouyssegur J (2008) Tumor cell metabolism: cancer’s Achilles’ heel. Cancer Cell 13: 472–482. 18538731 10.1016/j.ccr.2008.05.005

[13] Merker MP, Bongard RD, Kettenhofen NJ, Okamoto Y, Dawson CA (2002) Intracellular redox status affects transplasma membrane electron transport in pulmonary arterial endothelial cells. Am J Physiol Lung Cell Mol Physiol 282: L36–43. 11741813 10.1152/ajplung.00283.2001

[14] Alcain FJ, Buron MI, Villalba JM, Navas P (1991) Ascorbate is regenerated by HL-60 cells through the transplasmalemma redox system. Biochim Biophys Acta 1073: 380–385. 2009284 10.1016/0304-4165(91)90146-8

[15] del Castillo-Olivares A, Nunez de Castro I, Medina MA (2000) Dual role of plasma membrane electron transport systems in defense. Crit Rev Biochem Mol Biol 35: 197–220. 10907796 10.1080/10409230091169203

[16] Hyun DH, Hernandez JO, Mattson MP, de Cabo R (2006) The plasma membrane redox system in aging. Ageing Res Rev 5: 209–220. 16697277 10.1016/j.arr.2006.03.005

[17] Beyer RE, Segura-Aguilar J, Di Bernardo S, Cavazzoni M, Fato R, et al (1996) The role of DT-diaphorase in the maintenance of the reduced antioxidant form of coenzyme Q in membrane systems. Proc Natl Acad Sci U S A 93: 2528–2532. 8637908 10.1073/pnas.93.6.2528 PMC39831

[18] Mataix J, Manas M, Quiles J, Battino M, Cassinello M, et al (1997) Coenzyme Q content depends upon oxidative stress and dietary fat unsaturation. Mol Aspects Med 18 Suppl: S129–135 10.1016/s0098-2997(97)00019-8 9266514

[19] Navarro F, Navas P, Burgess JR, Bello RI, De Cabo R, et al (1998) Vitamin E and selenium deficiency induces expression of the ubiquinone-dependent antioxidant system at the plasma membrane. FASEB J 12: 1665–1673. 9837856 10.1096/fasebj.12.15.1665

[20] Villalba JM, Navarro F, Cordoba F, Serrano A, Arroyo A, et al (1995) Coenzyme Q reductase from liver plasma membrane: purification and role in trans-plasma-membrane electron transport. Proc Natl Acad Sci U S A 92: 4887–4891. 7761418 10.1073/pnas.92.11.4887 PMC41812

[21] Villalba JM, Navas P (2000) Plasma membrane redox system in the control of stress-induced apoptosis. Antioxid Redox Signal 2: 213–230. 11229527 10.1089/ars.2000.2.2-213

[22] Jaiswal AK (2004) Nrf2 signaling in coordinated activation of antioxidant gene expression. Free Radic Biol Med 36: 1199–1207. 15110384 10.1016/j.freeradbiomed.2004.02.074

[23] Johnson JA, Johnson DA, Kraft AD, Calkins MJ, Jakel RJ, et al (2008) The Nrf2-ARE pathway: an indicator and modulator of oxidative stress in neurodegeneration. Ann N Y Acad Sci 1147: 61–69. 19076431 10.1196/annals.1427.036 PMC2605641

[24] Son TG, Camandola S, Arumugam TV, Cutler RG, Telljohann RS, et al (2010) Plumbagin, a novel Nrf2/ARE activator, protects against cerebral ischemia. J Neurochem 112: 1316–1326. 20028456 10.1111/j.1471-4159.2009.06552.x PMC2819586

[25] Hyun DH, Mughal MR, Yang H, Lee JH, Ko EJ, et al (2010) The plasma membrane redox system is impaired by amyloid beta-peptide and in the hippocampus and cerebral cortex of 3xTgAD mice. Exp Neurol 225: 423–429. 20673763 10.1016/j.expneurol.2010.07.020 PMC2946538

[26] Raina AK, Takeda A, Nunomura A, Perry G, Smith MA (1999) Genetic evidence for oxidative stress in Alzheimer’s disease. Neuroreport 10: 1355–1357. 10363952 10.1097/00001756-199904260-00036

[27] Mattson MP (2004) Pathways towards and away from Alzheimer’s disease. Nature 430: 631–639. 15295589 10.1038/nature02621 PMC3091392

[28] Hyun DH, Kim J, Moon C, Lim CJ, de Cabo R, et al. (2011) The plasma membrane redox enzyme NQO1 sustains cellular energetics and protects human neuroblastoma cells against metabolic and proteotoxic stress. Age (Dordr). 10.1007/s11357-011-9245-1 PMC3312640 21487704

[29] Seow HA, Penketh PG, Belcourt MF, Tomasz M, Rockwell S, et al (2004) Nuclear overexpression of NAD(P)H:quinone oxidoreductase 1 in Chinese hamster ovary cells increases the cytotoxicity of mitomycin C under aerobic and hypoxic conditions. J Biol Chem 279: 31606–31612. 15155746 10.1074/jbc.M404910200

[30] Kelner GS, Lee M, Clark ME, Maciejewski D, McGrath D, et al (2000) The copper transport protein Atox1 promotes neuronal survival. J Biol Chem 275: 580–584. 10617654 10.1074/jbc.275.1.580

[31] Lee M, Hyun DH, Halliwell B, Jenner P (2001) Effect of overexpression of wild-type and mutant Cu/Zn-superoxide dismutases on oxidative stress and cell death induced by hydrogen peroxide, 4-hydroxynonenal or serum deprivation: potentiation of injury by ALS-related mutant superoxide dismutases and protection by Bcl–2. J Neurochem 78: 209–220. 11461956 10.1046/j.1471-4159.2001.00417.x

[32] Shim JH, Yoon SH, Kim KH, Han JY, Ha JY, et al (2011) The antioxidant Trolox helps recovery from the familial Parkinson’s disease-specific mitochondrial deficits caused by PINK1- and DJ-1-deficiency in dopaminergic neuronal cells. Mitochondrion 11: 707–715. 21664494 10.1016/j.mito.2011.05.013

[33] Bradford MM (1976) A rapid and sensitive method for the quantitation of microgram quantities of protein utilizing the principle of protein-dye binding. Anal Biochem 72: 248–254. 942051 10.1016/0003-2697(76)90527-3

[34] Hyun DH, Emerson SS, Jo DG, Mattson MP, de Cabo R (2006) Calorie restriction up-regulates the plasma membrane redox system in brain cells and suppresses oxidative stress during aging. Proc Natl Acad Sci U S A 103: 19908–19912. 17167053 10.1073/pnas.0608008103 PMC1750890

[35] Hyun DH, Hunt ND, Emerson SS, Hernandez JO, Mattson MP, et al (2007) Up-regulation of plasma membrane-associated redox activities in neuronal cells lacking functional mitochondria. J Neurochem 100: 1364–1374. 17250676 10.1111/j.1471-4159.2006.04411.x

[36] Vives-Bauza C, Yang L, Manfredi G (2007) Assay of mitochondrial ATP synthesis in animal cells and tissues. Methods Cell Biol 80: 155–171. 17445693 10.1016/S0091-679X(06)80007-5

[37] Zhou M, Diwu Z, Panchuk-Voloshina N, Haugland RP (1997) A stable nonfluorescent derivative of resorufin for the fluorometric determination of trace hydrogen peroxide: applications in detecting the activity of phagocyte NADPH oxidase and other oxidases. Anal Biochem 253: 162–168. 9367498 10.1006/abio.1997.2391

[38] French SA, Territo PR, Balaban RS (1998) Correction for inner filter effects in turbid samples: fluorescence assays of mitochondrial NADH. Am J Physiol 275: C900–909. 9730975 10.1152/ajpcell.1998.275.3.C900

[39] Li X, May JM (2002) Catalase-dependent measurement of H_2_O_2_ in intact mitochondria. Mitochondrion 1: 447–453. 16120297 10.1016/s1567-7249(02)00010-7

[40] Tahara EB, Navarete FD, Kowaltowski AJ (2009) Tissue-, substrate-, and site-specific characteristics of mitochondrial reactive oxygen species generation. Free Radic Biol Med 46: 1283–1297. 19245829 10.1016/j.freeradbiomed.2009.02.008

[41] Votyakova TV, Reynolds IJ (2004) Detection of hydrogen peroxide with Amplex Red: interference by NADH and reduced glutathione auto-oxidation. Arch Biochem Biophys 431: 138–144. 15464736 10.1016/j.abb.2004.07.025

[42] Janssen AJ, Trijbels FJ, Sengers RC, Smeitink JA, van den Heuvel LP, et al (2007) Spectrophotometric assay for complex I of the respiratory chain in tissue samples and cultured fibroblasts. Clin Chem 53: 729–734. 17332151 10.1373/clinchem.2006.078873

[43] Lyras L, Cairns NJ, Jenner A, Jenner P, Halliwell B (1997) An assessment of oxidative damage to proteins, lipids, and DNA in brain from patients with Alzheimer’s disease. J Neurochem 68: 2061–2069. 9109533 10.1046/j.1471-4159.1997.68052061.x

[44] Reznick AZ, Packer L (1994) Oxidative damage to proteins: spectrophotometric method for carbonyl assay. Methods Enzymol 233: 357–363. 8015470 10.1016/s0076-6879(94)33041-7

[45] Haefeli RH, Erb M, Gemperli AC, Robay D, Courdier Fruh I, et al. NQO1-dependent redox cycling of idebenone: effects on cellular redox potential and energy levels. PLoS One 6: e17963. 10.1371/journal.pone.0017963 PMC3069029 21483849

[46] Zhu L, Liu Z, Feng Z, Hao J, Shen W, et al (2010) Hydroxytyrosol protects against oxidative damage by simultaneous activation of mitochondrial biogenesis and phase II detoxifying enzyme systems in retinal pigment epithelial cells. J Nutr Biochem 21: 1089–1098. 20149621 10.1016/j.jnutbio.2009.09.006

[47] Jia Z, Zhu H, Misra HP, Li Y (2008) Potent induction of total cellular GSH and NQO1 as well as mitochondrial GSH by 3H-1,2-dithiole-3-thione in SH-SY5Y neuroblastoma cells and primary human neurons: protection against neurocytotoxicity elicited by dopamine, 6-hydroxydopamine, 4-hydroxy-2-nonenal, or hydrogen peroxide. Brain Res 1197: 159–169. 18234165 10.1016/j.brainres.2007.12.044 PMC2386954

[48] Zhu H, Jia Z, Strobl JS, Ehrich M, Misra HP, et al (2008) Potent induction of total cellular and mitochondrial antioxidants and phase 2 enzymes by cruciferous sulforaphane in rat aortic smooth muscle cells: cytoprotection against oxidative and electrophilic stress. Cardiovasc Toxicol 8: 115–125. 18607771 10.1007/s12012-008-9020-4

[49] Zhu H, Jia Z, Zhou K, Misra HP, Santo A, et al (2009) Cruciferous dithiolethione-mediated coordinated induction of total cellular and mitochondrial antioxidants and phase 2 enzymes in human primary cardiomyocytes: cytoprotection against oxidative/electrophilic stress and doxorubicin toxicity. Exp Biol Med (Maywood) 234: 418–429. 19176875 10.3181/0811-RM-340

[50] Du J, Daniels DH, Asbury C, Venkataraman S, Liu J, et al (2006) Mitochondrial production of reactive oxygen species mediate dicumarol-induced cytotoxicity in cancer cells. J Biol Chem 281: 37416–37426. 17040906 10.1074/jbc.M605063200

[51] Gonzalez-Aragon D, Ariza J, Villalba JM (2007) Dicoumarol impairs mitochondrial electron transport and pyrimidine biosynthesis in human myeloid leukemia HL-60 cells. Biochem Pharmacol 73: 427–439. 17123468 10.1016/j.bcp.2006.10.016

[52] Bey EA, Bentle MS, Reinicke KE, Dong Y, Yang CR, et al (2007) An NQO1- and PARP-1-mediated cell death pathway induced in non-small-cell lung cancer cells by beta-lapachone. Proc Natl Acad Sci U S A 104: 11832–11837. 17609380 10.1073/pnas.0702176104 PMC1913860

[53] Andreeva L, Crompton M (1994) An ADP-sensitive cyclosporin-A-binding protein in rat liver mitochondria. Eur J Biochem 221: 261–268. 8168515 10.1111/j.1432-1033.1994.tb18737.x

[54] James AM, Smith RA, Murphy MP (2004) Antioxidant and prooxidant properties of mitochondrial Coenzyme Q. Arch Biochem Biophys. 423: 47–56. 10.1016/j.abb.2003.12.025 14989264

[55] Barja G (1999) Mitochondrial oxygen radical generation and leak: sites of production in states 4 and 3, organ specificity, and relation to aging and longevity. J Bioenerg Biomembr 31: 347–366. 10665525 10.1023/a:1005427919188

[56] Kushnareva Y, Murphy AN, Andreyev A (2002) Complex I-mediated reactive oxygen species generation: modulation by cytochrome c and NAD(P)+ oxidation-reduction state. Biochem J 368: 545–553. 12180906 10.1042/BJ20021121 PMC1222999

[57] Muller FL, Liu Y, Van Remmen H (2004) Complex III releases superoxide to both sides of the inner mitochondrial membrane. J Biol Chem 279: 49064–49073. 15317809 10.1074/jbc.M407715200

[58] Panov AV, Andreeva L, Greenamyre JT (2004) Quantitative evaluation of the effects of mitochondrial permeability transition pore modifiers on accumulation of calcium phosphate: comparison of rat liver and brain mitochondria. Arch Biochem Biophys 424: 44–52. 15019835 10.1016/j.abb.2004.01.013

[59] Xie HR, Hu LS, Li GY SH-SY5Y human neuroblastoma cell line: in vitro cell model of dopaminergic neurons in Parkinson’s disease. Chin Med J (Engl) 123: 1086–1092. 20497720

[60] Lambert MP, Stevens G, Sabo S, Barber K, Wang G, et al (1994) Beta/A4-evoked degeneration of differentiated SH-SY5Y human neuroblastoma cells. J Neurosci Res 39: 377–385. 7533843 10.1002/jnr.490390404

[61] Datki Z, Papp R, Zadori D, Soos K, Fulop L, et al (2004) In vitro model of neurotoxicity of Abeta 1–42 and neuroprotection by a pentapeptide: irreversible events during the first hour. Neurobiol Dis 17: 507–515. 15571986 10.1016/j.nbd.2004.08.007

[62] Li Y, Duffy KB, Ottinger MA, Ray B, Bailey JA, et al. GLP-1 receptor stimulation reduces amyloid-beta peptide accumulation and cytotoxicity in cellular and animal models of Alzheimer’s disease. J Alzheimers Dis 19: 1205–1219. 10.3233/JAD-2010-1314 PMC2948479 20308787

[63] Elkon H, Melamed E, Offen D (2001) 6-Hydroxydopamine increases ubiquitin-conjugates and protein degradation: implications for the pathogenesis of Parkinson’s disease. Cell Mol Neurobiol 21: 771–781. 12043847 10.1023/A:1015160323009 PMC11533854

[64] Sadan O, Bahat-Stromza M, Barhum Y, Levy YS, Pisnevsky A, et al (2009) Protective effects of neurotrophic factor-secreting cells in a 6-OHDA rat model of Parkinson disease. Stem Cells Dev 18: 1179–1190. 19243240 10.1089/scd.2008.0411

[65] Li Y, Tweedie D, Mattson MP, Holloway HW, Greig NH Enhancing the GLP-1 receptor signaling pathway leads to proliferation and neuroprotection in human neuroblastoma cells. J Neurochem 113: 1621–1631. 10.1111/j.1471-4159.2010.06731.x PMC2912144 20374430

[66] Incerpi S, Fiore AM, De Vito P, Pedersen JZ (2007) Involvement of plasma membrane redox systems in hormone action. J Pharm Pharmacol 59: 1711–1720. 18053334 10.1211/jpp.59.12.0014

[67] Okamura T, Kurisu K, Yamamoto W, Takano H, Nishiyama M (2000) NADPH/quinone oxidoreductase is a priority target of glioblastoma chemotherapy. Int J Oncol 16: 295–303. 10639573 10.3892/ijo.16.2.295

[68] Ahn KS, Sethi G, Jain AK, Jaiswal AK, Aggarwal BB (2006) Genetic deletion of NAD(P)H:quinone oxidoreductase 1 abrogates activation of nuclear factor-kappaB, IkappaBalpha kinase, c-Jun N-terminal kinase, Akt, p38, and p44/42 mitogen-activated protein kinases and potentiates apoptosis. J Biol Chem 281: 19798–19808. 16682409 10.1074/jbc.M601162200

[69] Scott KA, Barnes J, Whitehead RC, Stratford IJ, Nolan KA (2011) Inhibitors of NQO1: identification of compounds more potent than dicoumarol without associated off-target effects. Biochem Pharmacol 81: 355–363. 20970406 10.1016/j.bcp.2010.10.011

[70] Patrick BA, Gong X, Jaiswal AK (2011) Disruption of NAD(P)H:quinone oxidoreductase 1 gene in mice leads to 20S proteasomal degradation of p63 resulting in thinning of epithelium and chemical-induced skin cancer. Oncogene 30: 1098–1107. 21042282 10.1038/onc.2010.491 PMC3307144

