# Supplementary figures and images for: Mitochondrial Function in Human Neuroblastoma Cells Is Up-Regulated and Protected by NQO1, a Plasma Membrane Redox Enzyme
Source: PLoS One. 2013 Jul 11;8(7):e69030. doi: 10.1371/journal.pone.0069030 (PMC3708898; doi:10.1371/journal.pone.0069030)

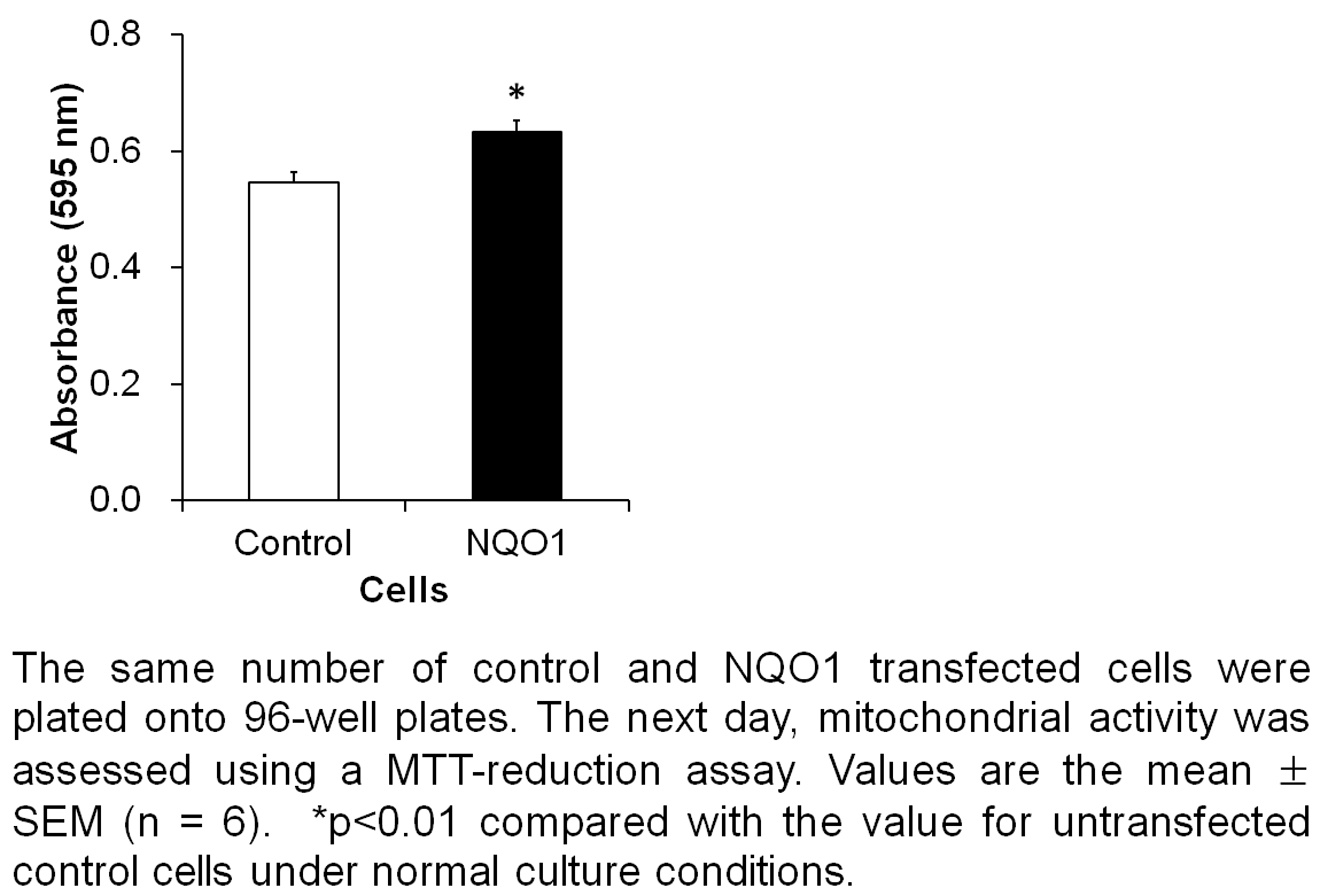

Supplement: Figure S1 — The same number of control and NQO1 transfected cells were plated onto 96-well plates. The next day, mitochondrial activity was assessed using a MTT-reduction assay. Values are the mean ± SEM (n = 6). *p<0.01 compared with the value for untransfected control cells under normal culture conditions. (TIF) [file pone.0069030.s001.tif]

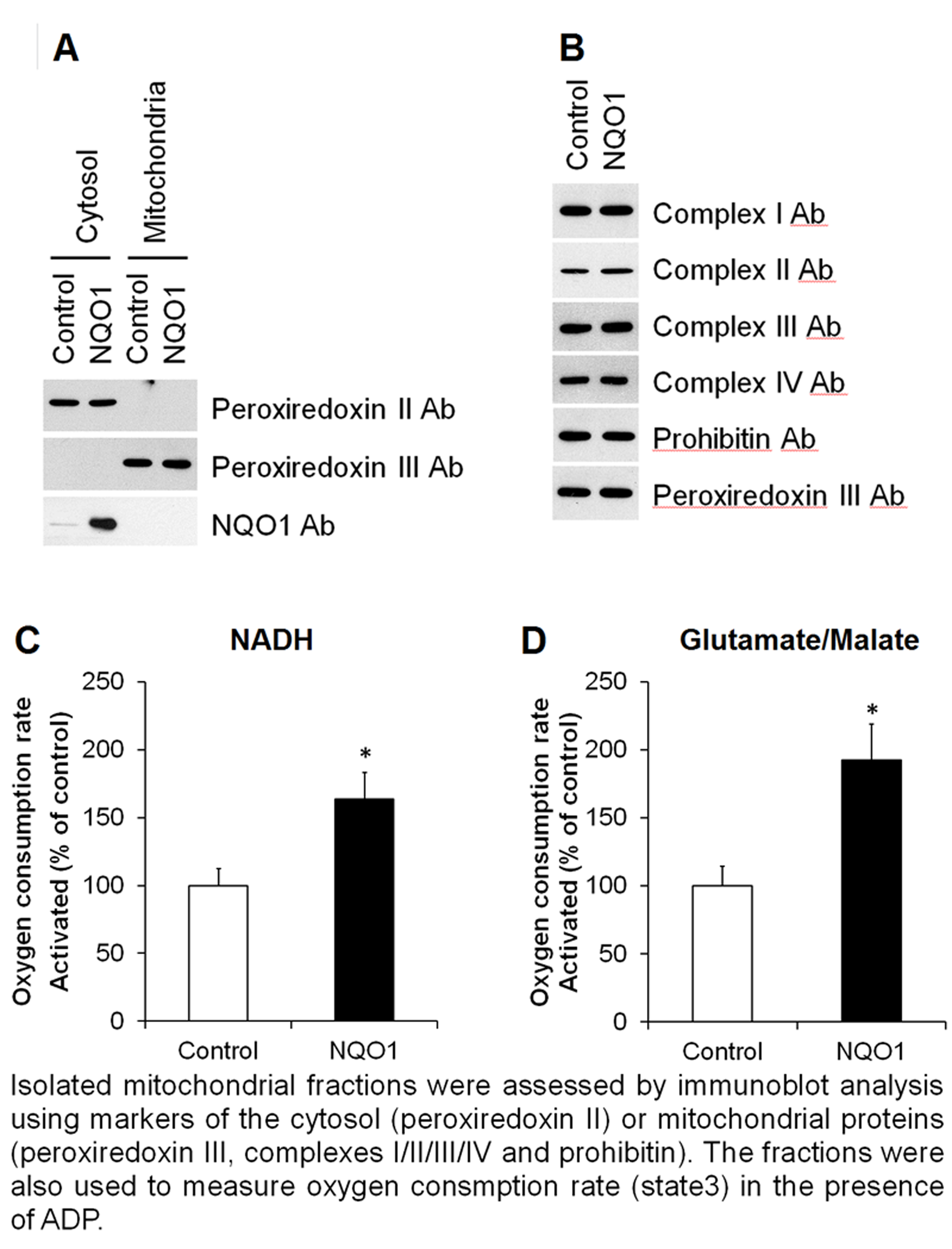

Supplement: Figure S2 — Isolated mitochondrial fractions were assessed by immunoblot analysis using markers of the cytosol (peroxiredoxin II) or mitochondrial proteins (peroxiredoxin III, complexes I/II/III/IV and prohibitin). The fractions were also used to measure oxygen consumption rate (state3) in the presence of ADP. (TIF) [file pone.0069030.s002.tif]
